# Leveraging feedback mechanisms to improve the quality of objective structured clinical examinations in Singapore: an exploratory action research study

**DOI:** 10.3352/jeehp.2025.22.28

**Published:** 2025-09-30

**Authors:** Han Ting Jillian Yeo, Dujeepa Dasharatha Samarasekera, Michael Dean

**Affiliations:** 1 Centre for Medical Education (CenMED), Yong Loo Lin School of Medicine, National University of Singapore, Singapore; 2 Graduate School of Education, College of Professional Studies, Northeastern University, Boston, MA, USA; The Catholic University of Korea, Korea

**Keywords:** Psychological feedback, Educational measurement, Objective structured clinical examination, Qualitative research, Singapore

## Abstract

**Purpose:**

Variability in examiner scoring threatens the fairness and reliability of objective structured clinical examinations (OSCEs). While examiner standardization exists, there is currently no structured, psychometric-informed, individualized feedback mechanism for examiners. This study explored the feasibility and perceived value of such a mechanism using an action research approach to co-design and iteratively refine examiner feedback reports.

**Methods:**

Two exploratory cycles were conducted between November 2023 and June 2024 with phase 4 OSCE examiners at the Yong Loo Lin School of Medicine. In cycle 1, psychometric analyses of examiner scoring for a phase 4 OSCE informed the design of individualized reports, which were evaluated through interviews. Revisions were made to the format of the report and implemented in cycle 2, where examiner responses were again collected. Data were analyzed thematically, supported by reflective logs and field notes.

**Results:**

Nine examiners participated in cycle 1 and 7 in cycle 2. In cycle 1, examiners highlighted challenges in interpreting complex terminology, leading to report refinements such as glossaries and visual graphs. In cycle 2, examiners demonstrated greater confidence in applying feedback, requested longitudinal reports, and shifted from initial resistance to reflective engagement. Across cycles, the reports improved credibility, neutrality, and examiner self-regulation.

**Conclusion:**

This exploratory study suggests that psychometric-informed feedback reports can facilitate examiner reflection and transparency in OSCEs. While the findings highlight feasibility and examiner acceptance, longitudinal delivery of feedback, collection of quantitative outcome data, and larger samples are needed to establish whether such reports improve scoring consistency and assessment fairness.

## Graphical abstract

[Fig f4-jeehp-22-28]

## Introduction

### Background

Clinical skills are fundamental to a clinician’s core duties [1]. Objective structured clinical examinations (OSCEs) were introduced to assess clinical competence, aiming to standardize and reduce variation in clinical assessments [2]. However, examiner scoring variability challenged this notion; one study reported up to a 16% change in pass/fail decisions with different examiner cohorts for the same OSCEs [3]. Students have cited factors such as gender, ethnicity, and personality as influences on scores, echoing faculty concerns that OSCEs remain subjective [4]. This is particularly critical in high-stakes assessments affecting students' progression or graduation. Such challenges are common globally as OSCEs are widely used to assess clinical skills [5]. Addressing examiner variability is therefore essential for upholding fairness and reliability. Research has suggested that structured feedback could mitigate variability by helping examiners recognize and adjust deviations from normative patterns [6,7]. While examiner training guidelines exist [8], little empirical evidence supports individualized, psychometric-informed feedback frameworks to promote reflective practice.

### Objectives

This study explored the feasibility and perceived value of an individualized examiner feedback report that leveraged psychometric analysis and structured reflection to enhance OSCE quality and fairness. The reports were iteratively co-designed and refined with examiners to examine how they might promote reflective practice and guide strategies for reducing examiner variability.

## Methods

### Ethics statement

The study has been approved by the Institutional Review Boards from both Northeastern University (CPS 22-11-26) and National University of Singapore (NUS-IRB-2023-954).

### Personal characteristics of research team

This study was part of the Doctor of Education program of the first author (J.H.T.Y.). Two authors (J.H.T.Y. and D.D.S.), as medical educationalists, provided in-depth insights into assessment processes. The first author (J.H.T.Y.) maintained a reflective log for reflexivity, while the third author (M.D.), as the doctoral supervisor, offered external guidance on methodology and direction.

### Theoretical framework and methodology: methodological orientation

The study employed the action research framework developed by Lewin [9], who believed that knowledge should be created from solving real-world problems. Action research provided a pragmatic lens to assess current practices, identify gaps, and propose possible actions with stakeholders. The cyclical nature of action research—gathering information, collecting, and analyzing data—enabled the creation of action steps that directly addressed practical needs. With limited evidence on psychometric-informed feedback, an exploratory approach was deemed appropriate for eliciting examiner perspectives, iteratively refining the intervention, and guiding future research. Reporting (Supplement 1) followed the Consolidated Criteria for Reporting Qualitative Research (COREQ) 32-item checklist [10].

### Context

The Yong Loo Lin School of Medicine at the National University of Singapore (NUS Medicine) offers a 5-year medical program using various assessment tools including OSCEs for evaluating clinical skills.

### Data collection

Examiners of phase 4 medical undergraduate OSCEs at NUS Medicine meeting the inclusion criteria were recruited via purposive email sampling from January to April 2024. Participation was voluntary. Nine examiners took part in cycle 1, with 7 returning for cycle 2. All were clinicians with extensive assessment experience, including question and standard setting, ranging from 1 to over 20 years. No consistent effects of experience, gender, or role on engagement with feedback reports or themes were observed.

Two authors (J.H.T.Y. and M.D.) developed a semi-structured interview guide (Supplement 2), iteratively revised during data collection to explore examiners’ views on the report’s clarity, usefulness, gaps, and reflections. The first author (J.H.T.Y.) conducted 30–45-minute Zoom interviews, which were audio-recorded and transcribed. Field notes captured contextual nuances and reflections, informing the iterative refinement of the feedback report.

Data saturation was monitored across interview cycles. By cycle 2, recurring themes indicated sufficient data to meet the study’s aims. Transcripts were not returned for participant review to minimize burden. Instead, the iterative interviews allowed clarification of emerging themes in subsequent discussions, ensuring accurate representation of participants’ perspectives.

### Data analysis

The first author (J.H.T.Y.) coded data using in vivo and descriptive methods. Reflective logs and peer debriefing with two authors (M.D. and D.D.S.) increased trustworthiness by challenging interpretations. Transcripts were analyzed iteratively after each cycle to ensure that emergent themes guided subsequent data collection.

## Results

### Developing the individualized feedback report

Feedback was designed following AMEE Guide No. 145 using examiners’ scores retrieved from the institution’s online marking portal after the phase 4 OSCEs [8]. A generalizability study using a “p×(i:s)” design was conducted via urGENOVA and mGENOVA software freely available from the Center for Advanced Studies in Measurement and Assessment at the University of Iowa [11]. This analysis identified potential measurement errors in OSCEs and estimated variance components across examiners, test items, stations, and candidates, assessing exam quality and reliability. Many-facets Rasch measurement (MFRM) using WINSTEPS software ver. 3.83.6 (https://www.winsteps.com/winsteps.htm) measured examiner stringency, item difficulty, candidate ability, and consistency of scores on a common scale [12]. These measures were incorporated into feedback reports.

**Fig. 1.**
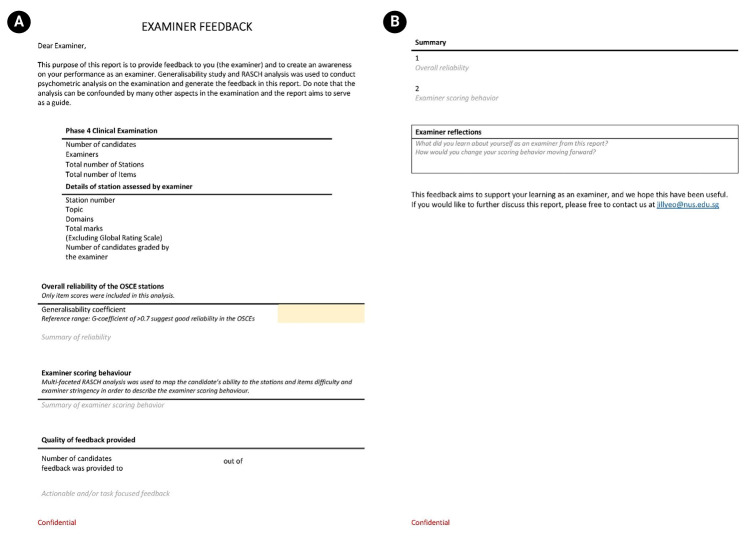
(A, B) First version of the examiner feedback report.

Between August and December 2023, a psychometric expert and the institution’s assessment committee reviewed the initial draft of the feedback report. They recommended a more narrative format and removal of technical data, such as fit statistics and variance components, to improve clarity and accessibility.International examples of examiner feedback guided the refinement of version 1 (Fig. 1).

### Cycle 1: Evaluation of version 1

Cycle 1 involved evaluating the initial version with examiners. Interview data revealed that the report was difficult to interpret due to statistical and educational jargon unfamiliar outside specialist fields. As Examiner 1 shared: “I guess it’ll be challenging to put this out to everyone who may not have the privilege of listening to all of you who are statisticians and psychometricians. If they are given this feedback, it may be difficult to interpret.”

In addition to concerns about accessibility, examiners expressed a strong desire for benchmarking to contextualize their scoring. As remarked by Examiner 3: “[If] I’m at a 50th percentile now, then it’s very clear to me that okay, I am on par, and what we want to be is in that band. But if I know I’m in the 40th percentile, then straightaway I know that I need some extra work …”

These findings highlighted the need for clearer explanations and comparative data.

### Cycle 2: Revision and evaluation of version 2

**Fig. 2.**
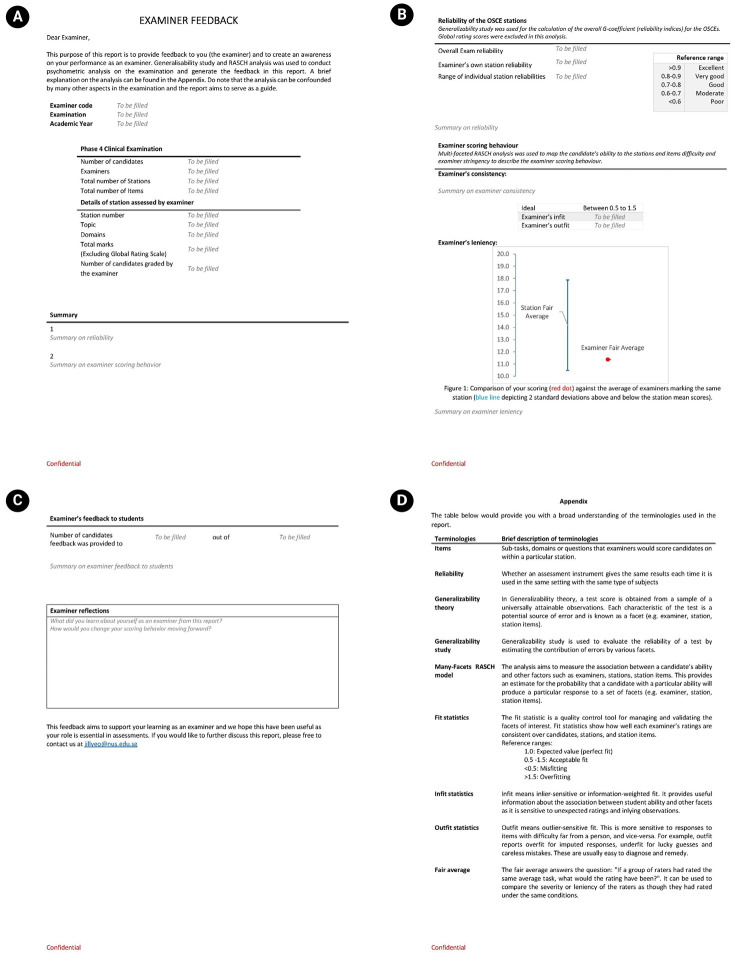
(A–D) Second version of the examiner feedback report.

In response, the report was revised (Fig. 2). To improve accessibility, an appendix was added to clarify complex terminologies. Examiners noted that the glossary improved accessibility, Examiner 2 stated: “There’s a glossary, so that really helps the people who are not familiar with the educational lingo, with some of the words.”

Graphical displays were added to strengthen benchmarking. Examiners could compare their fair averages with peers at the same station, offering a visual representation of relative stringency or leniency. Fair averages obtained through the MFRM enable comparisons of examiner severity or leniency as though all had rated under identical conditions. In addition, fit statistics were included to evaluate scoring consistency across students. Derived from the MFRM, these statistics indicated how consistently each examiner rated candidates, stations, and station items. Examiner 4 commented: “I think this is very concrete, very easy to understand because I’m marking, and I know what my score is. Then I know what generally other people’s scores are.”

### Cycle 2: Interpretation of a data-driven report

The inclusion of psychometric measures and graphs in the second cycle made examiners more aware of a systematic analysis in place aimed at quantifying their scoring behavior. Examiner 4 said: “The entire process was very rigorously evaluated after the exam; we do psychometrics of both students as well as the examiner. And it’s an iterative process, because you’re trying to see what can be improved.”

The psychometric measures provided in the revised report supported the overall narrative, making it easier for examiners to interpret and understand the feedback on their scoring patterns. Examiner 5 described: “I think statistics lend more oomph. The impression when I read the first one [...] it just felt like you’re just giving me, like, you know, sweeping comments. But when I saw the statistics, I realized that there were some computation and science to it.”

Quantitative measures were seen as objective and neutral, making the report acceptable and unbiased. This framing ensured that the feedback was constructive rather than accusatory, reducing defensiveness and promoting examiner acceptance. Examiner 6 explained: “Your report didn’t say that you are poor examiner, you’re not fair. And because of that next time, I will not call you.”

### Engagement in reflective practice

**Fig. 3.**
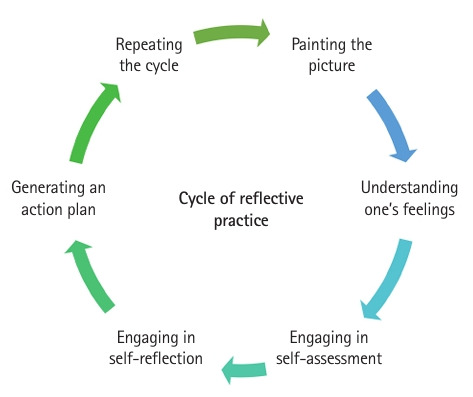
Cycle of reflective practice.

The delivery of feedback revealed stronger engagement in reflective practice, aligning with Schön’s concept of professionals surfacing implicit knowledge through experience [13]. Through the interviews, examiners demonstrated these steps: painting the picture, acknowledging their feelings, engaging in self-assessment and self-reflection, formulating an action plan, and repeating the cycle (Fig. 3).

Describing the report as “novel” and “niche,” examiners highlighted the absence of such feedback in the past. The report offered transparency on their scoring behaviors, as Examiner 4 remarked in cycle 1: “I think this feedback will be something that’s very useful to know exactly how I fare as an examiner because for the longest time I had no idea what I was doing after I did the exam.”

Version 2 of the feedback report enabled examiners to assess their performance against specific criteria, particularly after the inclusion of graphical displays for benchmarking. Examiner 6 remarked upon reviewing the second version of the report: “So, I’m a bit of a hawk [laughs], a bit hawkish!”

Self-reflection involved examining one’s thoughts, feelings, and behaviors to make more informed decisions. During interviews, examiners narrated their reflective process, recalling their experiences scoring medical undergraduates as they reviewed the report. Examiner 8 recounted in cycle 2: “When I think about it, how do I get this score? Then I can try to find a reason why I think that this score reflects where I really am in this area, for example. Did the score really reflect where I am?”

The shift in examiners’ future scoring behaviors between the first and second cycles was striking. Initially, all examiners asserted that the report would not affect their future scoring, displaying resistance, hesitation, and caution—rooted in longstanding practices and reluctance to change. Examiner 1 commented in cycle 1: “If you say that you tend to score lower than others, I suppose next time, I guess I’ll self-moderate. But again, I just want to be careful in not moderating just to get better feedback through this.”

In the second cycle, examiners who received feedback on inconsistencies, strictness, and leniency noted that the report offered guidance on how they might adjust their assessment practices in the future. Examiner 5 acknowledged the tendency to be stricter: “It also tells me that, if I want to move a little, I could be maybe a little bit more generous in my marking.”

Examiner 7, who exhibited more leniency, reflected on how this feedback might influence his future behavior: “I mean just looking at this. Then I think that I’m probably a bit more lenient than most people, but not unnecessarily, I mean, not unreasonably so. Just that next time I may not be so lenient. [laughs] [...] if I think there’s someone borderline. I may try to reflect it a bit more accurately.”

In the second cycle, all 7 examiners emphasized the need for ongoing access to feedback reports for all examiners. They highlighted regular feedback as essential for monitoring progress, evaluating performance, and deepening reflective practice. Recurring patterns in the reports reinforced their credibility, underscoring structured feedback as vital for accountability, professional development, and excellence. Examiner 5 opined: “If I had this feedback like 3 times in a year, and I was always on one end of the spectrum. Then it would give me pause to think about myself as an examiner.”

Engaging in reflective practice proved valuable for personal and professional growth, though certain factors influenced its effectiveness. Two examiners observed that small groups could respond defensively to perceived negative feedback, reducing their willingness to engage in self-reflection and self-assessment. Examiner 1 explained: “The only danger is that if somehow you get feedback and somebody gets a so-called negative, I mean negative in that sense, then either that person would not be invited again, or the person would turn down and not volunteer.”

However, this view was contradicted by others who felt that most examiners embodied a growth mindset and have an intrinsic desire for continuous improvement. Examiner 5 elaborated: “I want to know how I’m doing, and I always want to be better because ultimately I want to be a good and fair examiner.”

## Discussion

### Key results

Examiner variability affects assessment reliability, yet guidance on individualized, data-driven feedback is limited [3,4]. This exploratory study examined how psychometric-informed feedback reports could support examiner reflection in OSCEs. Across 2 action research cycles, examiners progressed from uncertainty to deeper self-assessment, with iterative refinements—glossaries, benchmarking, and graphics—enhancing credibility, neutrality, and usability.

### Interpretation

The findings are preliminary and descriptive. Clear, non-punitive feedback promoted acceptance and behavioral change. Longitudinal feedback supported monitoring, scoring refinement, and reflective practice. These findings suggest that institutions implementing similar feedback systems should prioritize longitudinal over one-time delivery, integrate psychometric analysis for objective insights, and encourage structured reflective practice using guided prompts and trend analysis.

### Comparison with previous studies

Most interventions focused on pre-OSCE examiner training such as prompting, scoring, and providing feedback to students [14]. In contrast, post-OSCE interventions typically provided structured feedback based on observed scores [6,7,15]. This study was novel in integrating psychometric techniques, such as generalizability study and MFRM, to quantify examiner leniency, consistency, and reliability, offering deeper self-assessment insights.

Moreover, although the reflective practice framework by Schön [13] is widely applied in student learning and clinical training, its role in examiner development is underexplored. Here, reflection-in-action and reflection-on-action were extended to assessment quality improvement, showing how structured feedback supports examiner self-regulation and professional growth.

### Limitations

This study had several limitations. First, the small and voluntary sample restricted the reliability and generalizability of the findings. Participants might have been more motivated and reflective than the wider examiner pool, potentially biasing the results.

The study did not measure quantitative changes in examiner variance or scoring consistency before and after the intervention. As such, the effectiveness of the feedback mechanism in reducing variability remains unproven and warrants further research with larger, representative samples and statistical analyses.

Third, the reliance on a single coder for qualitative analysis raises concerns about confirmability. While reflective logs and peer debriefing were employed to enhance trustworthiness, the absence of multiple coders or inter-coder checks remained a methodological weakness.

### Generalizability

Systemic changes fostered reflective practice, transparency, and accountability within a single institution. Although findings were from a single site, the feedback model’s principles are adaptable to other educational contexts, as OSCEs are widely used in medical education worldwide.

### Suggestions

This study introduced a scalable, data-driven approach to addressing examiner variability, moving beyond anecdotal or subjective training models. Future evaluation of longitudinal feedback delivery using advanced psychometric analyses can help determine the impact of feedback on examiner performance and scoring consistency. Incorporating pre- and post-intervention statistical analyses would provide more robust evidence of effectiveness.

### Conclusion

Structured, psychometric-informed feedback reports promote examiner reflection and fairer OSCE scoring. Iterative action research co-design enhanced usability and credibility. Institutions should combine longitudinal feedback with quantitative evaluation of scoring variability to strengthen assessment quality.

## Figures and Tables

**Figure f4-jeehp-22-28:**
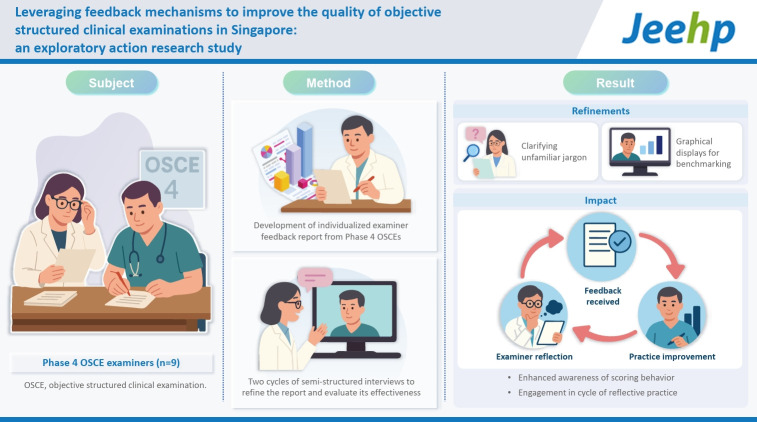


## Supplementary materials

Supplement 1. Consolidated criteria for reporting qualitative research (COREQ) item checklist.

Supplement 2. Interview protocol.

Supplement 3. Audio recording of the abstract.
