# Supplementary material for: Leveraging feedback mechanisms to improve the quality of objective structured clinical examinations in Singapore: an exploratory action research study
Source: J Educ Eval Health Prof. 2025 Sep 30;22:28. doi: 10.3352/jeehp.2025.22.28 (PMC12768547; doi:10.3352/jeehp.2025.22.28)
Supplement: Supplementary file 2 — Supplement 1. Consolidated criteria for reporting qualitative research (COREQ) item checklist. [file jeehp-22-28-suppl1.docx]

**Supplementary File 1**

**Semi-Structured Interview Protocol – First Cycle**

Interviewee (Title and Name): ______________________________________

RESEARCH QUESTION(S):

- What are the potential strategies that can be put in place to ensure fairness of OSCEs?
- How can examiner variability in the OSCEs be reduced in order to optimize the fairness in the OSCEs?

**Part I: Introductory Session (5-7 minutes):**

1. **Build rapport, describe the purpose of the interview, answer any questions.**
2. **Review Informed Consent Documents**
3. **Potential Script:**

You have been invited to speak with me today because you have been identified as someone who can share based on your experience as an OSCE examiner. My action research study focuses on how we can optimize our assessment practices to ensure fairness during the OSCEs for the medical undergraduates at NUS. One of the main themes which have emerged to have influenced the fairness in the OSCEs was the examiner scoring behavior. Because your responses are important and I want to make sure to capture everything you say, I would like to audio tape our conversation today. Do I have your permission to record this interview?

[if yes, thank the participant, let them know you may ask the question again as you start recording, and then turn on the recording equipment].

I will also be taking written notes. I can assure you that all responses will be confidential and only a pseudonym will be used when quoting from the transcripts. I will be the only one with access to the tapes which will be eventually destroyed after they are transcribed

Do you have any questions about the interview process or how your data will be used? This interview should last about 30 to 45minutes. During this time, I have several questions that I would like to cover. If time begins to run short, it may be necessary to interrupt you in order to push ahead and complete this line of questioning. Do you have any questions at this time?

**Part II: Interview**

1. Interviewee Background **(5 minutes):** To establish rapport and obtain background information about the participant.

- What is your role in NUS Medicine?
- What are your roles in teaching in NUS Medicine?
  - Which Phase are you teaching?
  - Which subjects are you teaching?
- What is your role in assessments in NUS Medicine? How long have you been in performing your role in assessments in NUS Medicine?
- How long have you been performing your role as an OSCE assessor?

1. Core Interview **(30 minutes):**

- Describe the challenges you faced as an OSCE examiner.
- How do you think these can be overcome?
- How can you be better supported?
- What is your understanding of assessment psychometrics (for example RASCH and Generalizability theory)?

I am now sharing with you an individualized examiner report that was generated from the Phase 4 OSCE conducted in AY23/24. You make take the next 5 to 10 minutes to review it.

| Value | - What were areas that were of value to you?   - Probing question:   Why were they of value to you?  Were the recommendations helpful for you?   - What were areas that were not of value to you?   - Probing question:   Why were they of value to you? |
| --- | --- |
| Clarity of report | - What areas do you think need more clarity? - How would you propose to improve the areas that require more clarity? |
| Gaps in report | - What additional information do you think would help the assessor’s performance? |
| Emotional reaction | - How did you feel when you were reviewing this report? - Why did you feel this way? |
| Ways forward | - What was your overall perspective on the performance of the assessment?   - Did the report allow you to benchmark your performance? - How would this influence your examination practice in future?   - Probing question:   Can you elaborate a bit more? |

*Before we conclude this interview, is there something about your experience as an examiner involved in undergraduate medicine assessments that we have not yet had a chance to discuss?*

**Semi-Structured Interview Protocol – Second Cycle**

Interviewee (Title and Name): ______________________________________

RESEARCH QUESTION(S):

- What are the potential strategies that can be put in place to ensure fairness of OSCEs?
- How can examiner variability in the OSCEs be reduced in order to optimize the fairness in the OSCEs?

**Part I: Introductory Session (5-7 minutes):**

1. **Build rapport, describe the purpose of the interview, answer any questions.**
2. **Review Informed Consent Documents**
3. **Potential Script:**

Thank you for agreeing to participate in a follow-up interview with regards to reviewing the feedback report. This interview should last about 20 to 30 minutes. During this time, I have several questions that I would like to cover. If time begins to run short, it may be necessary to interrupt you in order to push ahead and complete this line of questioning. Do you have any questions at this time?

Because your responses are important and I want to make sure to capture everything you say, I would like to audio tape our conversation today. Do I have your permission to record this interview?

[if yes, thank the participant, let them know you may ask the question again as you start recording, and then turn on the recording equipment].

I will also be taking written notes. I can assure you that all responses will be confidential and only a pseudonym will be used when quoting from the transcripts. I will be the only one with access to the tapes which will be eventually destroyed after they are transcribed

1. Core Interview **(20 minutes):**

I am sharing with you the revised version of the individualized examiner report based on the themes which had emerged from the first interview. Similar to the first version, this examiner report was generated from the Phase 4 OSCE conducted in AY23/24. You make take the next 5 to 10 minutes to review it.

| Comparison with old version | - What was something new that you learnt in the revised report?   - Was it of value to you?   - Was it not of value to you? - Which version do you prefer? And why? - Did this version provide greater clarity to you?   - What areas do you think needs more clarity? - How would you propose to improve the areas that require more clarity? |
| --- | --- |
| Domains in the report | - How do you understand reliability to be? Do you understand the values that are provided in the report? - How do you interpret your consistency as an examiner? - How do you understand your leniency / stringency as an examiner? - How do you interpret the comments given on your feedback to students? - Were the charts and values useful for you? - Was the glossary useful to you? |
| Gaps in report | - What additional information would you like to guide you in your scoring behavior? |
| Future directions | - Moving forward, would you like to receive more of such reports? - Do you think examiners should receive such reports? Why? |

*Before we conclude this interview, is there something about your experience as an examiner involved in undergraduate medicine assessments that we have not yet had a chance to discuss?*
