# Supplementary material for: Leveraging feedback mechanisms to improve the quality of objective structured clinical examinations in Singapore: an exploratory action research study
Source: J Educ Eval Health Prof. 2025 Sep 30;22:28. doi: 10.3352/jeehp.2025.22.28 (PMC12768547; doi:10.3352/jeehp.2025.22.28)
Supplement: Supplementary file 3 — Supplement 2. Interview protocol. [file jeehp-22-28-suppl2.docx]

Supplementary File 2

The Consolidated Criteria for Reporting Qualitative Studies (COREQ): 32-item checklist

| Topic | Item No. | Guide Questions/ Description | Notes |
| --- | --- | --- | --- |
| **Domain 1: Research team and reflexivity** | | | |
| Personal characteristics | | | |
| Interviewer/facilitator | 1 | Which author/s conducted the interview or focus group? | JY and DS, as medical educationalists, provided in-depth insights into assessment processes. MD, as the doctoral supervisor, offered external guidance on methodology and direction. |
| Credentials | 2 | What were the researcher’s credentials? E.g. PhD, MD | JY: MSc, EdD  DS: MD  MD: PhD |
| Occupation | 3 | What was their occupation at the time of the study? | JY: Medical educationalist and EdD candidate  DS: Senior director and medical educationalist  MD: Associate teaching professor |
| Gender | 4 | Was the researcher male or female? | JY is female. DS and MD are males. |
| Experience and training | 5 | What experience or training did the researcher have? |  |
| Relationship with participants | | | |
| Relationship established | 6 | Was a relationship established prior to study commencement? | JY and DS have worked with some participants through their involvements in assessment. MD has no contact with participants/ |
| Participant knowledge of the interviewer | 7 | What did the participants know about the researcher? e.g. personal goals, reasons for doing the research | Participants were aware that this was part of a doctoral work which aimed to reduce examiner variability. |
| Interviewer characteristics | 8 | What characteristics were reported about the interviewer/facilitator? e.g. Bias, assumptions, reasons and interests in the research topic | The research team had an interest in how data driven feedback could impact examiner variability. |
| **Domain 2: Study design** | | | |
| Theoretical framework | | | |
| Methodological orientation and Theory | 9 | What methodological orientation was stated to underpin the study? e.g. grounded theory, discourse analysis, ethnography, phenomenology, content analysis | The research team engaged in participatory action research. |
| Participant selection | | | |
| Sampling | 10 | How were participants selected? e.g. purposive, convenience, consecutive, snowball | Participants were selected using purposive sampling. The study specifically recruited examiners involved in the assessment of Phase Four medical undergraduates who met the inclusion criteria. |
| Method of approach | 11 | How were participants approached? e.g. face-to-face, telephone, mail, email | Participants were approached via email. |
| Sample size | 12 | How many participants were in the study? | A total of nine examiners participated in the study: Nine Phase Four clinical examiners were interviewed in the first cycle. Seven of these examiners participated again in the second cycle using the revised feedback report. |
| Non-participation | 13 | How many people refused to participate or dropped out? Reasons? | Of the 231 examiners invited, nine examiners agreed to participate in the study.  Two participants dropped out in the subsequent citing reasons of being busy with clinical work. |
| Setting | | | |
| Setting of data collection | 14 | Where was the data collected? e.g. home, clinic, workplace | Data were collected via Zoom™ video conferencing, with all interviews conducted online. |
| Presence of nonparticipants | 15 | Was anyone else present besides the participants and researchers? | There was no one else present. |
| Description of sample | 16 | What are the important characteristics of the sample? e.g. demographic data, date | In addition to their roles as examiners, participants who were all clinicians had extensive involvement in assessment, including question setting and standard setting. Their experience as examiners in the medical undergraduate program ranged from one year to over 20 years. |
| Data collection | | | |
| Interview guide | 17 | Were questions, prompts, guides provided by the authors? Was it pilot tested? | Please refer to the Interview Protocol |
| Repeat interviews | 18 | Were repeat interviews carried out? If yes, how many? | Yes, repeat interviews were carried out. Of the nine examiners interviewed in the first cycle, seven participated in a second round of interviews using the revised feedback report. |
| Audio/visual recording | 19 | Did the research use audio or visual recording to collect the data? | Yes, the research used **audio recording** to collect the data. |
| Field notes | 20 | Were field notes made during and/or after the interview or focus group? | JY maintained a reflective log throughout the study. |
| Duration | 21 | What was the duration of the interviews or focus group? | Interviews lasted between 30 to 45 minutes |
| Data saturation | 22 | Was data saturation discussed? | Data saturation was assessed throughout the iterative interview cycles. By the second cycle, recurring themes consistently emerged, indicating sufficient data to meet the study’s aims. |
| Transcripts returned | 23 | Were transcripts returned to participants for comment and/or correction? | Transcripts were not returned for participant correction to minimize burden, given clinicians' time constraints. Instead, the iterative interview process allowed emerging themes to be clarified in subsequent discussions, ensuring accurate representation of participants' perspectives. |
| **Domain 3: analysis and findings** | | | |
| Data analysis | | | |
| Number of data coders | 24 | How many data coders coded the data? | The data was coded by JY using an in vivo and descriptive coding approach to capture key patterns in participants' language. |
| Description of the coding tree | 25 | Did authors provide a description of the coding tree? | There was no description of a coding tree. |
| Derivation of themes | 26 | Were themes identified in advance or derived from the data? | Themes were derived in an inductive manner from the data. |
| Software | 27 | What software, if applicable, was used to manage the data? | No specialized software was used to manage the data. |
| Participant checking | 28 | Did participants provide feedback on the findings? | Participants provided feedback on the findings during the second interview. |
| Reporting | | | |
| Quotations presented | 29 | Were participant quotations presented to illustrate the themes/findings? Was each quotation identified? e.g. participant number | Key findings were supported with relevant quotes. |
| Data and findings consistent | 30 | Was there consistency between the data presented and the findings? | The themes and findings were derived from the data. |
| Clarity of major themes | 31 | Were major themes clearly presented in the findings? | Major themes were clearly numbered and titled. |
| Clarity of minor themes | 32 | Is there a description of diverse cases or discussion of minor themes? | Participants with alternative perspectives were discussed in the results section as well. |
